# Diabetes Mellitus Is an Independent Risk Factor for a Stiff Left Atrial Physiology After Catheter Ablation for Atrial Fibrillation

**DOI:** 10.3389/fcvm.2022.828478

**Published:** 2022-03-28

**Authors:** Moon-Hyun Kim, Hee Tae Yu, Yoon Jung Park, Tae-Hoon Kim, Boyoung Joung, Moon-Hyoung Lee, Hui-Nam Pak

**Affiliations:** Division of Cardiology, Department of Internal Medicine, Yonsei University Health System, Seoul, South Korea

**Keywords:** diabetes mellitus, atrial fibrillation, catheter ablation, pulmonary vascular resistance, stiff left atrium

## Abstract

**Background:**

Scar tissue formation after catheter ablation for atrial fibrillation (AF) may adversely affect the diastolic properties of the left atrium (LA), which can result in a stiff LA physiology in a small proportion of patients. In this study, we aimed to explore the relationship between diabetes mellitus and a stiff LA physiology after AF catheter ablation (AFCA).

**Methods:**

A total of 1,326 patients who underwent de novo AFCA, and baseline and 1-year follow-up echocardiographies were enrolled. After 1:3 propensity score (PS) matching for age, sex, and AF type, we compared 211 patients with DM with 633 patients without DM. A stiff LA physiology was defined as estimated pulmonary arterial pressure increase of >10 mmHg and a right ventricular systolic pressure of >35 mmHg at 1-year follow-up echocardiography. Pulmonary vascular resistance (PVR) was estimated using echocardiographic parameters.

**Results:**

Among the 844 PS-matched patients, a stiff LA physiology was observed in 32 patients (4.1%). The patients with DM showed a higher peak LA pressure (*p* < 0.001) and greater LA wall stress (*p* = 0.001) than did those without. A stiff LA physiology was independently associated with DM [Odds ratio (OR) = 2.39, 95% confidence interval (CI) 1.02-5.59, *p* = 0.045], empirical extra-pulmonary vein LA ablation (OR = 3.14, 95% CI 1.07–9.3, *p* = 0.038) and the ΔPVR (OR = 1.78, 95% CI 1.37–2.31, *p* < 0.001). The ΔPVR was independently associated with DM (β = 0.37, 95% CI 0.06-0.67, *p* = 0.020) and a stiff LA physiology (β = 1.40, 95% CI 0.70–2.10, *p* < 0.001). During the 38.8 ± 29.3months follow-up, the incidence of the clinical recurrence of AF was significantly higher in the patients with a stiff LA physiology than in those without (log rank *p* = 0.032).

**Conclusion:**

A stiff LA physiology was independently associated with DM because of the relatively small decrease in the PVR after AFCA in this population. The patients with a stiff LA physiology had worse rhythm outcomes after AFCA than those without.

## Introduction

Atrial fibrillation (AF) is a prevalent arrhythmia that increases morbidity and socioeconomic burden worldwide (1). Currently, AF catheter ablation (AFCA) is an effective rhythm control method for patients with AF (1). Various clinical benefits of AFCA have been reported, including reduced mortality in patients with heart failure (2), reduced risk of stroke and improvements in cognitive function (3).

However, AFCA is a destructive procedure that uses heating or freezing as an energy source (4) and inevitably leads to atrial tissue damage, resulting in necrosis, and scarring. Previous studies have reported that AFCA, particularly extra-pulmonary vein (PV) left atrium (LA) ablation, increases LA stiffness, and worsens post-ablation diastolic function. Repeated catheter ablation has also been reported to increase LA pressure and stiffness compared with the de novo procedure (5). Patients with a higher LA pressure or stiffness or greater wall stress had a higher recurrence rate after AFCA (6). Stiff LA syndrome was first reported as symptomatic pulmonary arterial hypertension caused by a decreased LA function after mitral valve surgery (7). Recently, it has been reported that stiff LA syndrome may develop after extensive AFCA (8). In previous studies, diabetes mellitus (DM) was reported as independent risk factors for stiff LA syndrome (9). To date, there is limited knowledge on the mechanism of stiff LA syndrome after AFCA, and the relationship between stiff LA syndrome and metabolic causes, such as DM, is unknown.

In this study, we applied the term “stiff LA physiology” and used previously reported echocardiographic parameters to define this condition (10). One-year follow-up parameters were used to investigate the incidence and clinical features of a stiff LA physiology in patients who underwent AFCA. The purpose of this study was to explore the association between DM and a stiff LA physiology. We also attempted to elucidate the mechanism of a stiff LA physiology using pulmonary vascular resistance (PVR) derived from echocardiographic findings (11).

## Materials and Methods

### Study Population

**Figure 1:**
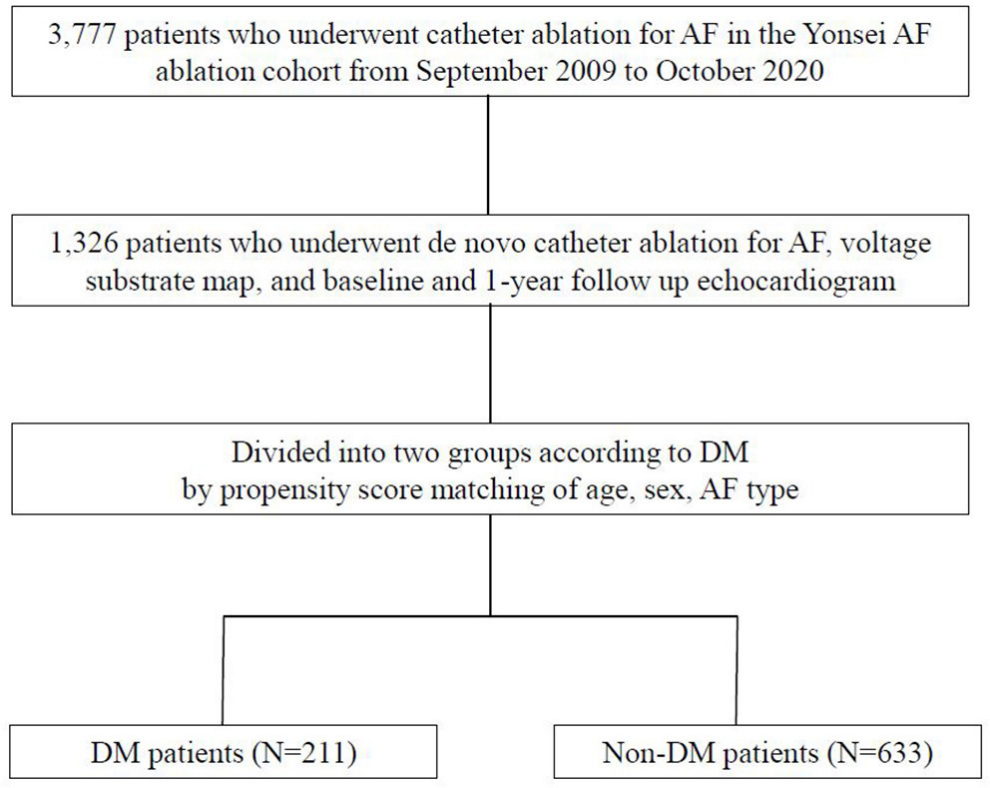
Study flow chart of patient enrollment. AF, atrial fibrillation; DM, diabetes mellitus.

The study protocol adhered to the principles of the Declaration of Helsinki and was approved by the institutional review board of the Yonsei University Health System. All patients provided written informed consent for inclusion in the Yonsei AF Ablation Cohort Database. Among 3,777 patients who underwent de novo AFCA in the Yonsei AF Ablation Cohort from September 2009 to October 2020, 1,326 patients who underwent voltage substrate mapping, baseline echocardiography, and 1-year follow-up echocardiography were enrolled in the study. DM was defined as HbA1c ≥ 6.5% or taking DM medication before the procedure (12). The study patients were divided into two groups according to the presence of DM via propensity score (PS) matching. After 1:3 PS matching for age, sex, and AF type, we compared 211 patients with DM with 633 patients without DM (Figure 1). The exclusion criteria of this study were as follows: (1) permanent AF refractory to electrical cardioversion; (2) AF with rheumatic valvular disease; (3) previous cardiac surgery with concomitant AF surgery or AFCA; (4) unmeasurable voltage map during sinus rhythm owing to frequent re-initiation of AF; and (5) no transthoracic echocardiography at baseline or 1 year later.

### Electrophysiological Mapping and Radiofrequency Catheter Ablation

Three-dimensional (3D) electroanatomical mapping (NavX; St. Jude Medical, Inc., Minnetonka, MN) using a circumferential PV mapping catheter (Lasso; Biosense-Webster Inc., Diamond Bar, CA) through a long sheath (Schwartz left 1; St. Jude Medical, Inc.) was performed. Pulmonary venography was performed after trans-septal punctures using a pigtail catheter. The 3D geometry of both the LA and PV was merged using the NavX system and then generated with 3D spiral computed tomographic (CT) images. Systemic anticoagulation with intravenous heparin was achieved to maintain an activated clotting time of 350-400s during the procedure. An open-irrigated tip catheter (Celsius; Johnson & Johnson Inc., Diamond Bar, CA; NaviStar ThermoCool, Biosense Webster Inc.; ThermoCool SF, Biosense Webster Inc.; ThermoCool SmartTouch, Biosense Webster Inc.; Coolflex, St. Jude Medical, Inc.; 30–35 W; 47°C; FlexAbility, St. Jude Medical, Inc.; and Tacti-Cath, St. Jude Medical, Inc.) was used for AFCA. All patients underwent a de novo procedure involving circumferential PV isolation (CPVI). Most patients (94.8%) received a cavotricuspid isthmus (CTI) block during the procedure unless there was an atrioventricular conduction disease. We conducted an additional linear ablation including a roof line, a posterior inferior line, and an anterior line, especially in the patients with persistent AF. Left lateral isthmus ablation, right atrial ablation, and complex fractionated electrogram ablation were performed in a minority of the patients at the operator's discretion. We defined extra-PV LA ablation as an additional linear ablation with or without complex fractionated electrogram ablation following CPVI. The procedure ended when there was no immediate recurrence of AF within 10 min after cardioversion with isoproterenol infusion (5–10 mcg/min).

### Echocardiographic and Cardiac Computed Tomographic Evaluations

All patients underwent transthoracic echocardiography at baseline and at the 1-year follow-up. The LA diameter, left ventricular (LV) ejection fraction (LVEF), LV mass index (LVMI), peak trans mitral flow velocity (E), and peak septal mitral annular velocity (Em) on tissue Doppler echocardiography were measured in accordance with the American Society of Echocardiography guidelines (13). Retrograde systolic tricuspid flow was obtained from the apical four-chamber view to measure the peak tricuspid pressure drop using continuous-wave Doppler. The LV outflow tract (LVOT) diameter and velocity-time integral (VTI) were measured from the parasternal long axis view and apical three-chamber view using pulsed-wave Doppler. PVR was estimated using the following equation: PVR = pulmonary arterial mean pressure (PAMP) echo–pulmonary capillary wedge pressure (PCWP)/cardiac output (CO) echo (11). PAMP echo was calculated as follows: Pulmonary arterial systolic pressure (PASP) echo × 0.61 + 2 mmHg. PCWP echo was calculated as follows: 1.24 × E/Em + 1.9 mmHg (14). Stroke volume (SV) and CO echo were estimated at the LVOT as follows: SV = (LVOT diameter/2)^2^ × LVOT VTI and CO echo = SV × heart rate, respectively (15). These parameters were defined as the PVR-related parameters. The delta value was calculated as follows: delta (Δ) = value at the 1-year follow-up echocardiography–value at the baseline echocardiography.

Three-dimensional spiral CT (64 Channel, Light Speed Volume CT, Philips, Brilliance 63; Amsterdam, the Netherlands) was performed to define the PV anatomy. The 3D spiral CT images of the LA were analyzed using an image processing workstation (Aquarius; TeraRecon, Inc., Foster City, CA).

### LA Pressure, LA Wall Thickness, and LAW Stress Measurement

Intracardiac electrograms and hemodynamic measurements were recorded using the Prucka Cardio Lab electrophysiology system (General Electric Medical Systems, Inc., Milwaukee, WI). For catheter access to the LA, a trans-septal puncture approach was used. During AFCA, the LA pressure was measured during sinus rhythm after the trans-septal puncture using a 6-F pigtail catheter (A&A Medical Device, Inc., Gyeonggi-do, Republic of Korea) that was inserted into the LA through a long sheath (Schwartz left 1; St. Jude Medical, Inc.). When the initial rhythm was AF, we measured the LA pressure during sinus rhythm after terminating the AF via internal cardioversion (2–10 J biphasic shocks, Lifepak12; Physiocontrol, Ltd., Redmond, WA), followed by a 3-min waiting period to allow for recovery from atrial stunning from cardioversion (5, 16). We analyzed the peak LA pressure (LAPpeak; v wave), LA nadir pressure (LAPnadir; x wave), and LA mean pressure (LAPmean). These parameters have been defined and calculated in our previous study (17).

We developed a customized software (AMBER, Laonmed Inc., Seoul, Republic of Korea) that measured the LAW thickness by applying Laplace's equation to the cardiac CT images. The LAW thickness was calculated as a numerical streamline connecting the endocardium and epicardium using the Euler method after solving the vector field with Laplace's equation, the partial differential equation in the 3D space (18). Thereafter, the mean LAW thickness was used as a parameter to calculate the LAW-stress. LAW-stress (dyn/cm^2^) was calculated using the law of Laplace [s = (P × r)/2 h (s, wall stress; P, pressure; r, radius; h, wall thickness)] (19). The peak LA pressure during sinus rhythm was directly measured during AF, and the LA radius was defined as half of the LA anteroposterior (AP) diameter on transthoracic echocardiography. Therefore, LAW-stress was calculated using the following equation: LAW-stress = (peak LA pressure × LA AP diameter)/(4 × LAW thickness). It was expressed in dyn/cm^2^ (1 mmHg = 1,333 dyn/cm^2^).

### Post-ablation Management and Follow-Up

The patients were instructed to visit the outpatient clinic at 1, 3, 6, and 12 months and then every 6 months thereafter or whenever symptoms occurred after RFCA. Electrocardiography was performed at every visit. Twenty-four-hour Holter monitoring was performed at 3, 6, and 12 months and every 6 months thereafter according to the 2012 Heart Rhythm Society/European Heart Rhythm Association/European Cardiac Arrhythmia Society Expert consensus statement guidelines (20). The patients who experienced symptoms of palpitations underwent Holter/event-monitor examinations to investigate the possibility of arrhythmia recurrence. AF recurrence was defined as any episode of atrial tachycardia (AT) or AF lasting for more than 30s. All electrocardiographic documentations of AF recurrence after a 3-month blanking period were classified as clinical recurrence.

### Statistical Analysis

Continuous variables were expressed as means ± standard deviations and compared using Student's *t*-test. Categorical variables were reported as counts (percentages) and compared using the chi-square or Fisher's exact test. Logistic regression analysis was used to investigate the variables related to a stiff LA physiology and DM. Linear regression analysis was used to investigate the variables related to the ΔPVR. The variables with *p*-values of <0.05 in the univariate analysis were selected for the multivariate analysis. Kaplan–Meier analysis with the log-rank test was used to analyze the probability of freedom from AF/AT recurrence after AFCA. Statistical significance was set at *p*-values of <0.05. The Statistical Package for the Social Sciences version 25.0 for Windows (IBM Corp., Armonk, NY) and R software version 3.6.2 (The R Foundation for Statistical Computing, Vienna, Austria) were used for the data analysis.

## Results

### Clinical Characteristics of the Patients With DM and a Stiff LA Physiology

1,326 patients were included for analysis and 211 patients were diagnosed with DM (15.9%). The baseline characteristics according to the presence of DM before and after PS matching are shown in Table 1. A total of 844 patients (male: 69.0%) PS-matched for age, sex, and AF type had an average age of 64.0 ± 8.7 years, and 58.6% had paroxysmal AF. The patients with DM had more comorbidities, such as hypertension (*p* < 0.001) and vascular disease (*p* < 0.001) than those without DM. The patients with DM showed a higher LA pressure and greater peak (*p* < 0.001) and LAW stress (*p* = 0.001) than did those without. There was no significant difference in performance of extra-PV LA ablation or CTI ablation between the two groups (Table 1). Among the 844 patients, a stiff LA physiology was observed in 32 patients (4.1%). The prevalence of DM was higher in the patients with a stiff LA physiology than in those without (*p* = 0.037). The peak LA pressure (*p* = 0.010) and LAW stress (*p* = 0.024) were also higher in the patients with a stiff LA physiology than in those without (Supplementary Table 1).

**Table 1:** Baseline characteristics according to the presence of DM.

	**Before propensity score matching (*****N*** **= 1,326)**			**After propensity score matching (*****N*** **= 844)**		
	**DM (*N* = 211)**	**Non-DM (*N* = 1,115)**	***P*** **-value**	**DM (*N* = 211)**	**Non-DM (*N* = 633)**	***P*** **-value**
**Clinical variables**						
Age, years	64.2 ± 8.8	58.8 ± 10.8	<0.001	64.2 ± 8.8	64.1 ± 8.7	0.911
Paroxysmal AF, %	121 (57.3)	722 (64.6)	0.051	121 (57.3)	374 (59.1)	0.687
Male, %	147 (69.7)	762 (68.2)	0.687	147 (69.7)	435 (68.7)	0.864
Body mass index, kg/m^2^	25.4 ± 3.0	24.8 ± 3.0	<0.001	25.4 ± 3.0	24.8 ± 2.8	<0.001
CHA_2_DS_2_VASc score	3.5 ± 1.6	1.6 ± 1.4	0.003	3.5 ± 1.6	2.0 ± 1.5	0.013
Congestive heart failure, %	28 (13.3)	169 (15.1)	0.528	28 (13.3)	96 (15.2)	0.575
Hypertension, %	161 (76.3)	471 (42.1)	<0.001	161 (76.3)	320 (50.6)	<0.001
Stroke, %	39 (18.5)	141 (12.6)	0.028	39 (18.5)	90 (14.2)	0.151
Vascular disease, %	56 (26.5)	114 (10.2)	<0.001	56 (26.5)	89 (14.1)	<0.001
**3D computed tomography**						
LA volume/BSA, mL/m^2^	87.1 ± 24.5	87.9 ± 31.1	0.688	87.1 ± 24.5	90.2 ± 24.9	0.125
Pericardial fat volume, mL	125.9 ± 55.7	110.3 ± 53.9	<0.001	125.9 ± 55.7	116.5 ± 54.3	0.035
**Catheter ablation**						
Ablation time, sec	4991.2 ± 1510.8	4910.2 ± 1645.2	0.508	4991.2 ± 1510.8	4979.8 ± 1632.0	0.929
Fluoroscopic time, min	37.9 ± 13.7	37.7 ± 14.5	0.843	37.9 ± 13.7	36.6 ± 13.6	0.231
Procedure time, min	185.9 ± 46.3	184.8 ± 49.7	0.774	185.9 ± 46.3	185.7 ± 49.5	0.970
Pulmonary vein ablation, %	211 (100.0)	1,118 (100.0)	–	211 (100.0)	633 (100.0)	–
Extra PV LA ablation, %	86 (41.0)	448 (40.1)	0.818	86 (41.0)	241 (38.1)	0.513
CTI, %	199 (94.8)	1,067 (95.6)	0.587	199 (94.8)	615 (97.2)	0.124
**LA related parameter**						
LA pressure, peak, mmHg	24.9 ± 10.5	22.1 ± 9.6	<0.001	24.9 ± 10.5	21.9 ± 9.5	<0.001
LA voltage	1.4 ± 0.7	1.3 ± 0.7	0.023	1.4 ± 0.7	1.4 ± 0.7	0.797
LA wall thickness	1.9 ± 0.4	2.0 ± 0.3	0.466	1.9 ± 0.4	2.0 ± 0.3	0.074
LA wall stress	193.6 ± 111.5	164.1 ± 95.2	0.001	193.6 ± 111.5	162.0 ± 94.3	0.001

*Data are presented as mean ± SD for continuous variables and as proportions for categorical variables. DM, diabetes mellitus; AF, atrial fibrillation; LA, left atrium; BSA, body surface area; CTI, cavo-tricuspid isthmus; PV, pulmonary vein*.

### Echocardiographic Characteristics in the Patients With a Stiff LA Physiology and DM

**Figure 2:**
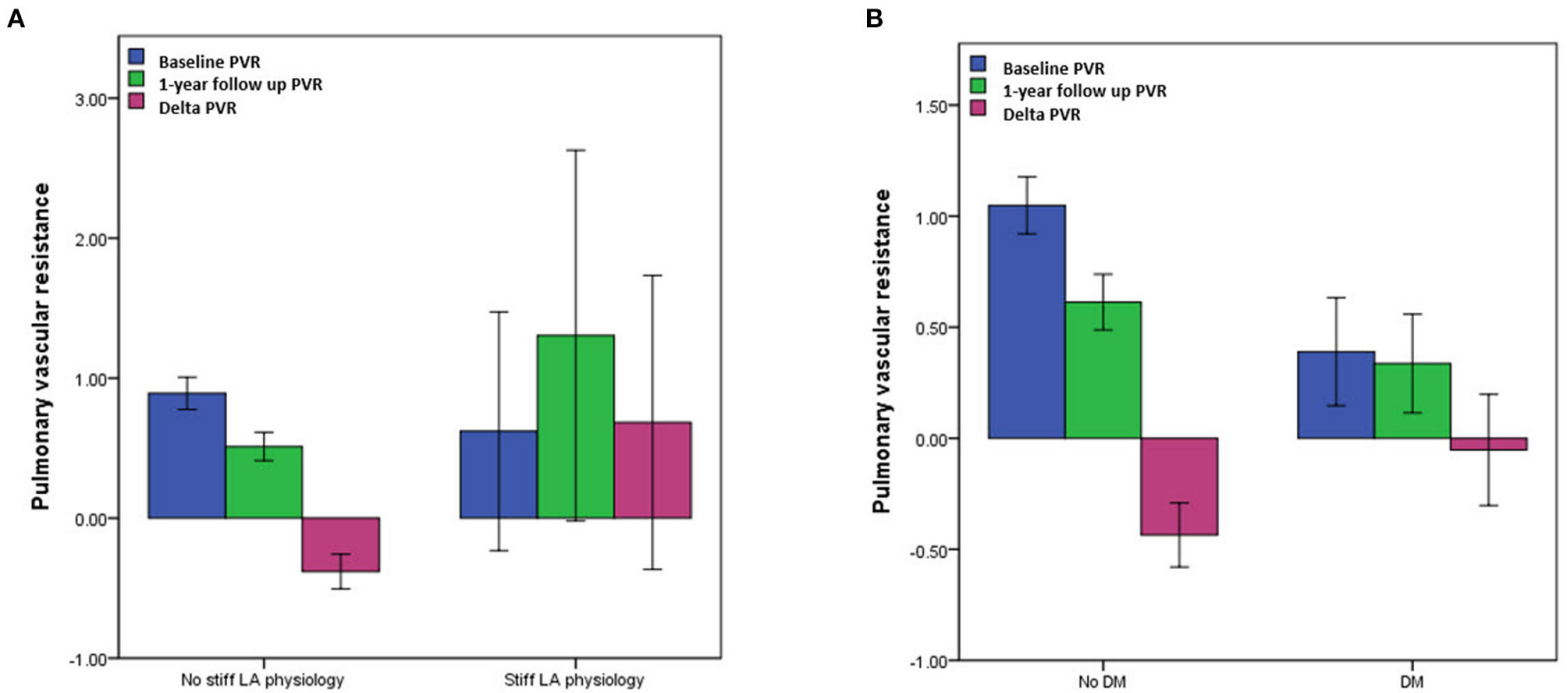
Comparisons of pulmonary vascular resistance (PVR) **(A)** according to the presence of a stiff LA physiology and **(B)** DM. PVR, pulmonary vascular resistance; LA, left atrium; DM, diabetes mellitus.

The echocardiographic characteristics according to the presence of a stiff LA physiology are shown in Table 2. There was no significant different in the parameters at baseline. At 1-year follow-up echocardiography, the patients with a stiff LA physiology after AFCA showed a larger LA diameter (*p* = 0.001) and higher E/Em (*p* = 0.001) and LVMI (*p* = 0.001) than did those without. Like the echocardiographic findings, there was no significant difference in the PVR-related parameters at baseline. However, at 1-year follow-up, the PAMP (*p* < 0.001) and PCWP (*p* = 0.001) were higher in the patients with a stiff LA physiology than in those without. Moreover, the patients with a stiff LA physiology showed a higher ΔPVR (*p* < 0.001) than did those without (Figure 2A).

**Table 2:** Echocardiographic characteristics according to the presence of a stiff LA physiology.

	**Baseline**			**1-year follow up**			**Delta value**		
	**Stiff LA physiology** **(*N* = 32)**	**No stiff LA physiology** **(*N* = 812)**	***p*** **-value**	**Stiff LA physiology** **(*N* = 32)**	**No stiff LA physiology** **(*N* = 812)**	***p*** **-value**	**Stiff LA physiology** **(*N* = 32)**	**No stiff LA physiology** **(*N* = 812)**	***p*** **-value**
**Echocardiographic findings**									
LA diameter, mm	43.5 ± 5.5	42.3 ± 6.0	0.263	42.7 ± 5.1	39.1 ± 5.7	0.001	−0.8 ± 4.5	−3.2 ± 4.7	0.005
LVEF, %	63.1 ± 7.6	62.7 ± 8.6	0.837	66.0 ± 10.7	64.8 ± 7.5	0.368	3.0 ± 7.1	2.0 ± 7.8	0.510
E/Em	12.2 ± 4.6	10.9 ± 4.4	0.085	17.7 ± 10.1	11.2 ± 4.7	0.001	5.3 ± 7.4	0.3 ± 3.9	0.001
RVSP, mmHg	27.8 ± 7.2	27.2 ± 6.9	0.639	44.8 ± 8.4	26.0 ± 6.0	<0.001	17.0 ± 4.3	−0.3 ± 17.6	<0.001
LVMI, g/m^2^	98.7 ± 18.2	96.5 ± 23.3	0.619	109.0 ± 23.8	96.1 ± 21.6	0.001	8.0 ± 21.9	−0.3 ± 17.6	0.013
**PVR related parameter**									
Stroke volume	53.1 ± 17.2	57.8 ± 19.7	0.187	68.7 ± 23.3	61.9 ± 17.6	0.114	15.5 ± 14.5	4.1 ± 21.4	<0.001
Heart rate, bpm	69.3 ± 16.5	66.7 ± 13.0	0.431	67.2 ± 17.7	71.5 ± 10.5	0.243	−0.9 ± 14.1	5.4 ± 13.3	0.079
Cardiac output	3.7 ± 1.2	3.9 ± 1.5	0.562	4.3 ± 1.4	4.4 ± 1.2	0.624	0.9 ± 1.3	0.5 ± 1.5	0.369
PAMP, mmHg	19.0 ± 4.4	18.6 ± 4.2	0.639	29.3 ± 5.1	17.8 ± 3.6	<0.001	10.4 ± 2.6	−0.8 ± 3.9	<0.001
PCWP, mmHg	17.1 ± 5.7	15.4 ± 5.4	0.085	23.9 ± 12.5	15.8 ± 5.8	0.001	6.6 ± 9.2	0.3 ± 4.8	0.001
PVR	0.5 ± 1.5	0.9 ± 1.7	0.303	1.7 ± 3.4	0.4 ± 1.5	0.604	1.1 ± 1.3	−0.5 ± 1.7	<0.001

*Data are presented as mean ± SD for continuous variables. LA, left atrium; LVEF, left ventricular ejection fraction; E/Em, the ratio of the early diastolic mitral inflow velocity (E) to the early diastolic mitral annular velocity (Em); RVSP, right ventricular systolic pressure; LVMI, left ventricular mass index; PVR, pulmonary vascular resistance; PAMP, pulmonary artery mean pressure; PCWP, pulmonary capillary wedge pressure*.

The echocardiographic characteristics according to the presence of DM are presented in Supplementary Tables 3, 4. While there was no significant difference in the LA diameter, the LVEF, right ventricular systolic pressure (RVSP), and E/Em (*p* < 0.001) were significantly higher in the patients with DM than in those without a baseline. The ΔPVR was also significantly higher in the patients with DM than in those without (*p* = 0.012) (Figure 2B).

### Association of a Stiff LA Physiology With DM and the ΔPVR

We investigated the association between a stiff LA physiology and DM using multivariate logistic regression analysis and linear regression analysis. In the adjusted model, a stiff LA physiology was independently associated with DM [OR = 2.39 (1.02–5.59), *p* = 0.045], the pericardial fat volume [OR = 1.01 (1.00–1.02), *p* = 0.004], empirical extra-PV LA ablation [OR = 3.14 (1.07–9.3), *p* = 0.038], and the ΔPVR [OR = 1.78 (1.37–2.31), *p* < 0.001]. We analyzed two multivariate models separately because the 1-year follow-up PVR and ΔPVR had multicollinearity. A stiff LA physiology was also associated with the 1-year follow-up PVR [OR = 1.69 (1.25–2.28), *p* = 0.001] (Table 3). To evaluate the contribution of diabetes to stiff LA physiology, an additional multivariate logistic analysis excluding patients of extra PV LA ablation group was performed. As a result, in this subgroup, stiff LA physiology also showed an independent association with 1-year follow-up PVR or ΔPVR (Supplementary Table 5). The multivariate linear regression analysis revealed that the ΔPVR was independently associated with DM [β = 0.37 (0.06–0.67), *p* = 0.020], the peak LA pressure [β = −0.02 (-0.03–0.00), *p* = 0.034], and a stiff LA physiology [β = 1.40 (0.70–2.10), *p* < 0.001] in the adjusted model (Table 4). The multivariate logistic regression analysis revealed that DM was independently associated with hypertension [OR = 2.25 (1.47–3.43), *p* < 0.001], vascular disease [OR = 1.78 (1.10–2.90), *p* = 0.020], and the ΔPVR [OR = 1.03 (1.01–1.05), *p* = 0.001] in the adjusted model (Supplementary Table 6).

**Table 3:** Logistic regression analysis of the stiff LA physiology in the patients.

	**Univariate analysis**		**Multivariate analysis**		**Multivariate analysis**	
	**OR (95% CI)**	***P*** **-value**	**OR (95% CI)**	***P*** **-value**	**OR (95% CI)**	***P*** **-value**
Age	1.042 (0.999–1.088)	0.058				
Male	0.854 (0.406–1.798)	0.678				
Paroxysmal AF	0.355 (0.169–0.746)	0.006	1.088 (0.378–3.134)	0.876	0.881 (0.302–2.575)	0.817
Body mass index	1.052 (0.935–1.183)	0.397				
Diabetes mellitus	2.122 (1.029–4.374)	0.042	2.935 (1.278–6.742)	0.011	2.386 (1.019–5.587)	0.045
Hypertension	0.969 (0.475–1.975)	0.931				
Congestive heart failure	2.000 (0.877–4.559)	0.099				
Stroke	0.563 (0.169–1.877)	0.350				
Vascular disease	1.945 (0.881–4.296)	0.100				
LA diameter	1.034 (0.975–1.095)	0.263				
LVEF	1.004 (0.963–1.047)	0.837				
E/Em	1.059 (0.992–1.130)	0.087				
LA volume/BSA	1.013 (0.999–1.026)	0.060				
Pericardial fat volume	1.008 (1.002–1.014)	0.007	1.010 (1.003–1.017)	0.006	1.011 (1.003–1.018)	0.004
LA pressure, peak^*^	1.061 (1.027–1.097)	<0.001				
LA voltage^*^	0.389 (0.205–0.738)	0.004				
LA wall stress^*^	1.004 (1.001–1.007)	0.005	1.002 (0.999–1.006)	0.153	1.003 (0.999–1.006)	0.144
Extra PV LA ablation	2.731 (1.316–5.664)	0.007	3.494 (1.190–10.259)	0.023	3.144 (1.067–9.262)	0.038
Extra PV trigger	0.318 (0.043–2.377)	0.264				
Baseline PVR	0.939 (0.757–1.164)	0.564				
1-year follow up PVR	1.395 (1.117–1.742)	0.003	1.689 (1.251–2.281)	0.001		
Delta PVR	1.141 (1.176–1.767)	<0.001			1.782 (1.372–2.314)	<0.001

* *LA wall stress was included in the multivariate analysis due to multicollinearity among three variables. Two multivariate models were separately presented because 1-year follow up PVR and delta PVR had a multicollinearity to each other. LA, left atrium; AF, atrial fibrillation; LVEF, left ventricular ejection fraction; E/Em, the ratio of the early diastolic mitral inflow velocity (E) to the early diastolic mitral annular velocity (Em); BSA, body surface area; PV, pulmonary vein; PVR, pulmonary vascular resistance*.

**Table 4:** Linear regression analysis of the ΔPVR in the patients.

	**Univariate analysis**		**Multivariate analysis**	
	**B (95% CI)**	***P*** **-value**	**B (95% CI)**	***P*** **-value**
Age	−0.010 (−0.025–0.004)	0.158		
Male	0.061 (−0.211–0.333)	0.660		
Paroxysmal AF	−0.047 (−0.302–0.207)	0.715		
Body mass index	0.023 (−0.020–0.067)	0.294		
Diabetes mellitus	0.383 (0.096–0.671)	0.009	0.365 (0.057–0.673)	0.020
Hypertension	0.069 (−0.185–0.323)	0.594		
Congestive heart failure	−0.162 (−0.533–0.210)	0.394		
Stroke	0.014 (−0.341–0.369)	0.937		
Vascular disease	−0.036 (−0.366–0.293)	0.829		
LA diameter	−0.017 (−0.038–0.004)	0.122		
LVEF	0.008 (−0.007–0.023)	0.287		
LA volume/BSA	−0.006 (−0.011–0.000)	0.034	−0.005 (−0.011–0.000)	0.057
Pericardial fat volume	0.000 (−0.002–0.003)	0.742		
LA pressure, peak	−0.016 (−0.030–−0.003)	0.018	−0.015 (−0.029–−0.001)	0.034
LA voltage	0.049 (−0.139–0.237)	0.609		
LA wall stress	−0.001 (−0.003–0.000)	0.097		
Stiff LA physiology	1.065 (0.427–1.702)	0.001	1.402 (0.701–2.103)	<0.001
Extra PV LA ablation	0.076 (−0.183–0.335)	0.564		
Extra PV trigger	−0.060 (−0.488–0.367)	0.781		

*PVR, pulmonary vascular resistance; AF, atrial fibrillation; LA, left atrium; LVEF, left ventricular ejection fraction; BSA, body surface area; PV, pulmonary vein*.

### Clinical Recurrence of AF After RFCA and a Stiff LA Physiology

**Figure 3:**
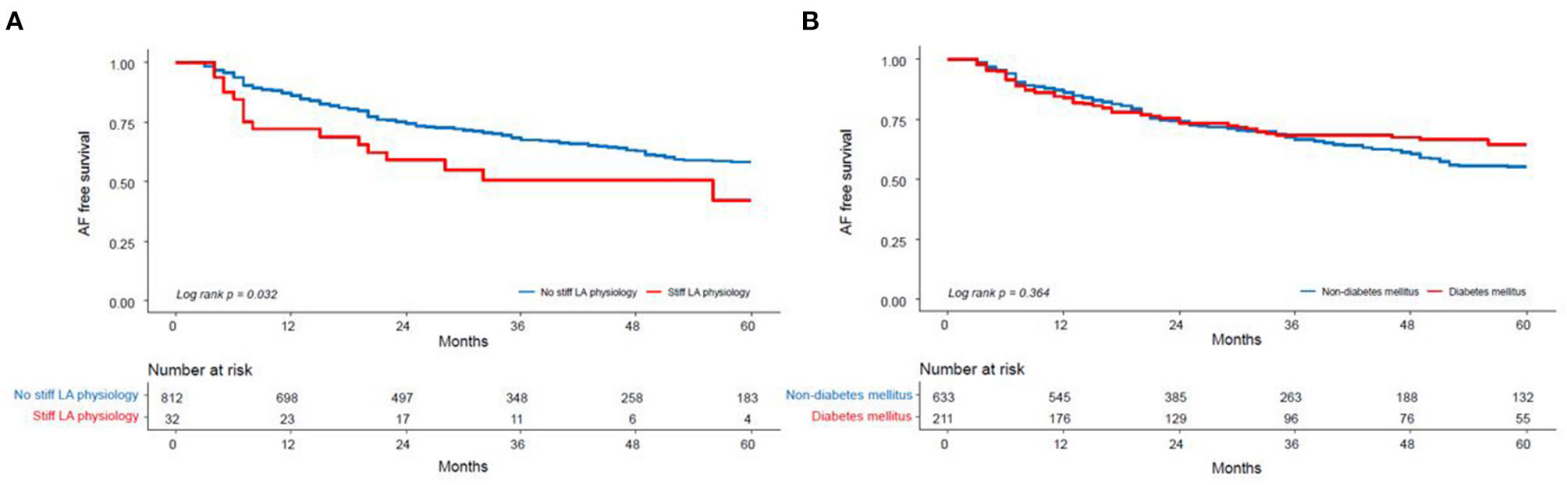
Kaplan–Meier analysis of the clinical recurrence of AF after AFCA **(A)** according to the presence of a stiff LA physiology and **(B)** DM. LA, left atrium; DM, diabetes mellitus.

During the 38.8 ± 29.3 month follow-up, the incidence of the clinical recurrence of AF was significantly higher in the patients with a stiff LA physiology than in those without (log rank *p* = 0.032) (Figure 3A, Supplementary Table 7). There was no difference in this incidence between the patients with and without DM (log rank *p* = 0.364) (Figure 3B).

## Discussion

Our study investigated the association between a stiff LA physiology after AFCA and DM using echocardiographic estimated PVR. We found that a stiff LA physiology was independently associated with DM, the ΔPVR, and empirical extra-PV LA ablation after adjustment for age, sex, and AF type. Compared with the patients without DM, a relatively small decrease in the PVR was observed in the patients with DM after AFCA, which may explain the mechanistic association between DM and a stiff LA physiology. Although the incidence of a stiff LA physiology was low, the clinical recurrence of AF after AFCA was associated with the presence of a stiff LA physiology in our study population.

### Stiff LA Physiology After AFCA

Stiff LA syndrome was first described in a patient who developed pulmonary artery hypertension with dyspnea after mitral valve replacement (7). Recently, the concept of stiff LA syndrome has recently been applied to patients who have undergone AFCA. The main clinical findings are dyspnea, congestive heart failure, pulmonary hypertension, and large v waves recorded on PCWP or LA pressure tracings in the absence of marked mitral regurgitation (21). Operators tend to create more ablation lesions to reduce the AF recurrence, but extensive ablation lesions usually result in more scar formation in the LA. Moreover, recent clinical trials have revealed no additional benefit of linear ablation or electrogram-guided ablation compared with a CPVI alone (22). In our recent study, extra-PV LA ablation markedly increased the LA pressure and worsened the diastolic function more than CPVI alone; however, there was no difference in the symptoms (5). In addition, we recently conducted a study on a stiff LA physiology defined using the RVSP. Similar to previous findings, DM, a low mean LA voltage, and extra-PV LA ablation were identified as risk factors (4).

### Association Between a Stiff LA Physiology and DM

In this study, patients who developed a stiff LA physiology showed an increased PVR after AFCA. There were no differences in the echocardiographic findings or PVR-related parameters at baseline. Among the patients with a stiff LA physiology, the SV increased, while the heart rate decreased after the procedure. There was no difference found in the CO between the two groups. Conversely, the PAMP and PCWP significantly increased in the patients with a stiff LA physiology. In particular, the increase in the PAMP was greater than that in the PCWP, which resulted in an increase in the PVR. Generally, the LA chamber is characterized by high compliance. It serves an atrial mechanical function through atrial contraction and as a reservoir by maintaining a low LA pressure during atrial relaxation and filling periods (23). Atrial contraction represents a wave in the LA pressure. Subsequent atrial relaxation shows a drop in the x wave, indicating early atrial filling in the PV, followed by an increase in the LA pressure and simultaneous LV contraction. As ventricular contraction continues, the RV also contracts, and the SV enters the LA through the pulmonary circulation and creates a v wave with passive filling (24). In a stiff LA physiology, LA compliance is reduced by atrial scarring and late systole is pronounced, which increases the v wave and PCWP. This can lead to pulmonary vascular remodeling, RV dysfunction, and increased pulmonary artery pressure. Previous studies have reported elevations in the E/Em reflecting diastolic dysfunction in patients with a stiff LA physiology (5). LV diastolic dysfunction increases the pulmonary artery pressure and RVSP and is attributed to a stiff LA physiology.

We found that a stiff LA physiology was independently associated with DM and the ΔPVR, and found an association between DM and the ΔPVR. In the comparison between the patients with and without DM, both groups showed a decrease in PVR after the procedure; however, the decrease in the PVR was smaller in the patients with DM. Regardless of DM, all patients showed a decrease in PVR after AFCA. Among the factors involved in estimated PVR, CO increased, PAMP decreased, and PCWP increased after AFCA. CO is affected by SV and HR, and as previously reported that HR increases in sinus rhythm after AFCA (25). Some studies showed that CO increases after AFCA (26). In addition, we previously reported an observation of increased LA pressure after AFCA (5). For the reasons described above, in the current analysis, it appears that the estimated PVR decreased after the procedure. However, the baseline PVR was significantly lower in the patients with DM, which resulted in a smaller ΔPVR. The reason why the baseline PVR was smaller in the patients with DM is that diastolic dysfunction occurs frequently among them, which increases the E/Em and PCWP (27). DM can lead to LA remodeling due to LA subendocardial fibrosis, oxidative stress, inflammation, and increased renin-angiotensin-aldosterone system activity as well as LV diastolic dysfunction (28). LA remodeling can increase the PCWP and decrease the PVR. Another study found that DM and hyperglycemia decreased pulmonary artery compliance and increased the RV afterload and RV remodeling in patients with pulmonary artery hypertension (29).

### Study Limitations

This study had several limitations. First, this was a single-center, observational, retrospective cohort study. Herein, we included highly selective patients who underwent de novo AFCA at a tertiary hospital. The change in the PVI technique was not clearly reflected as it had a long recruitment period of 11 years. Second, the definition of stiff LA syndrome included the patient's symptoms; however, we could not obtain data on the symptoms and thereby used the concept of a stiff LA “physiology” rather than “syndrome.” We defined a stiff LA physiology using the RVSP on echocardiography. In previous studies, LA stiffness was evaluated using the peak LA pressure, large v wave pressure, and LA pulse pressure (5, 9, 10). Since there is no gold standard method, LA stiffness was defined using several methods, and the relevant results of each study may be different. Thus, generalization of the results should be considered carefully. Third, the PVR was estimated using the echocardiographic findings. Patients without adequate pre- and post-procedural echocardiographic data were excluded. Potential confounding factors, such as age, comorbidities, medication, heart rate, and rhythm, may also play a role in post-procedural echocardiography. Fourth, we measured the LA pressure during sinus rhythm after cardioversion in the patients with early AF rhythm status. We waited for at least 3 min for the LA pressure to stabilize; however, it was difficult to rule out an effect on the LA after shock. Fifth, since we identified DM before the procedure, data regarding mean blood glucose levels, DM control, and period after diagnosis of DM were not available in this study. Further studies on DM control status including medication use, mean glucose level and stiff LA physiology will be needed. Despite the above limitations, we sought to evaluate the association of DM and a stiff LA physiology after AFCA in the current analysis, and by raising awareness of stiff LA physiology especially in diabetic populations, clinicians can have more attention to treatment options and prognosis for these patients.

## Conclusion

A stiff LA physiology was independently associated with DM because of the relatively small decrease in the PVR after AFCA in this population. The patients with a stiff LA physiology had worse rhythm outcomes after AFCA than in those without.

## Data Availability Statement

The original contributions presented in the study are included in the article/Supplementary Material, further inquiries can be directed to the corresponding author.

## Ethics Statement

The studies involving human participants were reviewed and approved by the Institutional Review Board of the Yonsei University Health System. The patients/participants provided their written informed consent to participate in this study. Written informed consent was obtained from the individual(s) for the publication of any potentially identifiable images or data included in this article.

## Author Contributions

M-HK contributed to the conception and design of the work, interpretation of data, and drafting of the manuscript. HY and H-NP contributed to the conception and design of the work and critical revision of the manuscript. YP, T-HK, BJ, and M-HL contributed to the conception and design of the work and revision of the manuscript. All authors read and approved the manuscript before its submission.

## Funding

This work was supported by grants [HI21C0011] from the Ministry of Health and Welfare and a grant [NRF-2020R1A2B5B01001695] from the Basic Science Research Program run by the National Research Foundation of Korea, which is funded by the Ministry of Science, ICT & Future Planning. This study was also supported by a Severance Hospital Research fund for Clinical excellence (SHRC) (C-2021-0019) and a new faculty research seed money grant of Yonsei University College of Medicine for 2021 (2021-32-0043).

## Conflict of Interest

The authors declare that the research was conducted in the absence of any commercial or financial relationships that could be construed as a potential conflict of interest.

## Publisher's Note

All claims expressed in this article are solely those of the authors and do not necessarily represent those of their affiliated organizations, or those of the publisher, the editors and the reviewers. Any product that may be evaluated in this article, or claim that may be made by its manufacturer, is not guaranteed or endorsed by the publisher.
