# Supplementary material for: Diabetes Mellitus Is an Independent Risk Factor for a Stiff Left Atrial Physiology After Catheter Ablation for Atrial Fibrillation
Source: Front Cardiovasc Med. 2022 Mar 28;9:828478. doi: 10.3389/fcvm.2022.828478 (PMC8995895; doi:10.3389/fcvm.2022.828478)
Supplement: Supplementary file 1 [file Data_Sheet_1.PDF]

Supplementary material

Supplementary Table 1. Baseline characteristics according to the presence of a stiff LA physiology

	**Before propensity score matching (N=1326)**			**After propensity score matching (N=844)**		
	**Stiff LA physiology (N=48)**	**No stiff LA physiology (N=1278)**	**P value**	**Stiff LA physiology (N=32)**	**No stiff LA physiology (N=812)**	**P value**
**Clinical variables**						
Age, years	63.2±9.3	59.5±10.7	0.017	67.0±8.0	64.0±8.7	0.057
Paroxysmal AF, %	18 (37.5)	822 (64.3)	<0.001	11 (34.4)	484 (59.6)	0.004
Male, %	32 (66.7)	874 (68.4)	0.801	21 (65.6)	561 (59.1)	0.678
Body mass index, kg/m2	25.5±3.4	24.9±3.0	0.131	25.4±3.1	25.0±2.8	0.398
CHA_2DS_2VASc score	2.3±1.8	1.9±1.6	0.128	2.8±1.9	2.4±1.7	0.187
Congestive heart failure, %	11 (22.9)	186 (14.6)	0.110	8 (25.0)	116 (14.3)	0.093
Hypertension, %	25 (52.1)	607 (47.5)	0.532	18 (56.3)	463 (57.0)	0.931
Diabetes mellitus, %	13 (27.1)	198 (15.5)	0.031	13 (40.6)	198 (24.4)	0.037
Stroke, %	5 (10.4)	175 (13.7)	0.515	3 (9.4)	126 (15.5)	0.344
Vascular disease, %	10 (20.8)	160 (12.5)	0.091	9 (28.1)	136 (16.7)	0.094
**3D computed tomography**						
LA volume/BSA, mL/m^2	100.7±29.1	87.4±30.1	0.004	98.0±22.6	89.1±24.9	0.060
Pericardial fat volume, mL	139.1±64.8	111.9±53.8	0.006	145.2±67.5	117.8±54.0	0.033
**Catheter ablation**						
Ablation time, min	5833.0±1989.7	4894.6±1596.7	0.002	6006.9±2014.7	4942.0±1570.9	0.006
Fluoroscopic time, min	43.8±15.7	37.5±14.2	0.003	42.5±13.7	36.7±13.6	0.018
Procedure time, min	213.0±52.7	184.1±48.6	<0.001	214.5±55.5	184.6±48.1	0.001
Pulmonary vein ablation, %	48 (100.0)	1278 (100.0)	-	32 (100.0)	812 (100.0)	-
Extra PV LA ablation, %	32 (66.7)	501 (39.3)	<0.001	20 (62.5)	307 (37.9)	0.005
CTI, %	48 (100.0)	1215 (95.3)	0.124	32 (100.0)	782 (96.4)	0.276
**LA related parameter**						
LA pressure, peak, mmHg	26.3±12.6	22.4±9.6	0.050	29.1±13.0	22.4±9.6	0.010
LA voltage	1.0±0.6	1.3±0.7	<0.001	1.1±0.6	1.4±0.7	0.003
LA wall thickness	1.9±0.3	2.0±0.3	0.423	1.9±0.3	2.0±0.3	0.278
LA wall stress	204.5±114.5	167.3±97.7	0.018	225.3±121.3	167.5±98.1	0.024

* Data are presented as mean ± SD for continuous variables and as proportions for categorical variables.

+ DM, diabetes mellitus; AF, atrial fibrillation; LA, left atrium; LVEF, left ventricular ejection fraction; E/Em, the ratio of the early diastolic mitral inflow velocity (E) to the early diastolic mitral annular velocity (Em); RVSP, right ventricular systolic pressure; LVMI, left ventricular mass index; BSA, body surface area; CTI, cavo-tricuspid isthmus

Supplementary Table 2. Hemodynamic characteristics according to the presence of a stiff LA physiology before propensity score matching

	Baseline			1-year follow up			Delta value		
	Stiff LA physiology (N=48)	No stiff LA physiology (N=1278)	p-value	Stiff LA physiology (N=48)	No stiff LA physiology (N=1278)	p-value	Stiff LA physiology (N=48)	No stiff LA physiology (N=1278)	p-value
**Echocardiographic findings**									
LA diameter, mm	44.3±5.8	41.8±6.3	0.007	44.1±6.4	38.9±6.0	<0.001	-0.7±4.5	-2.8±4.6	0.003
LVEF, %	62.8±7.7	62.7±8.7	0.931	65.2±9.5	64.6±7.4	0.597	2.7±7.6	1.7±8.0	0.388
E/Em	11.5±4.4	10.6±4.8	0.177	15.9±9.4	10.5±4.6	<0.001	5.4±7.3	0.2±3.8	<0.001
RVSP, mmHg	27.5±6.7	27.0±7.1	0.639	44.3±7.2	25.5±5.9	<0.001	16.5±4.6	-1.4±6.4	<0.001
LVMI, g/m^2	97.6±18.2	94.5±22.3	0.371	107.9±21.8	93.7±22.2	<0.001	7.1±21.3	-0.4±17.8	0.006
**PVR related parameter**									
Stroke volume	53.6±17.1	58.6±20.3	0.092	69.7±34.6	61.8±17.6	0.124	16.1±34.1	3.2±21.9	<0.001
Heart rate, bpm	70.1±17.7	67.4±12.9	0.425	67.3±16.1	71.2±10.9	0.043	-1.3±14.5	4.8±13.6	0.043
Cardiac output	3.8±1.1	4.0±1.3	0.230	4.4±2.4	4.3±1.2	0.386	0.6±2.4	0.3±1.5	0.255
PAMP, mmHg	18.8±4.1	18.5±4.3	0.639	29.0±4.4	17.6±3.6	<0.001	10.3±2.6	-0.9±4.1	<0.001
PCWP, mmHg	16.2±5.5	15.0±5.9	0.177	21.6±11.6	15.0±5.7	<0.001	5.6±8.1	0.0±4.9	<0.001
PVR	0.8±2.2	0.9±1.7	0.856	1.7±3.4	0.6±1.5	0.028	0.9±2.6	-0.3±1.6	0.003

* Data are presented as mean ± SD for continuous variables.

+ LA, left atrium; LVEF, left ventricular ejection fraction; E/Em, the ratio of the early diastolic mitral inflow velocity (E) to the early diastolic mitral annular velocity (Em); RVSP, right ventricular systolic pressure; LVMI, left ventricular mass index; PVR, pulmonary vascular resistance; PAMP, pulmonary artery mean pressure; PCWP, pulmonary capillary wedge pressure.

Supplementary Table 3. Hemodynamic characteristics according to the presence of DM before propensity score matching

	Baseline			1-year follow up			Delta value		
	DM (N=211)	Non-DM (N=1118)	p-value	DM (N=211)	Non-DM (N=1118)	p-value	DM (N=211)	Non-DM (N=1118)	p-value
**Echocardiographic findings**									
LA diameter, mm	43.0±6.0	41.7±6.3	0.005	40.6±5.5	38.8±6.2	<0.001	-2.2±4.9	-2.8±4.6	0.042
LVEF, %	62.0±8.9	62.9±8.7	0.197	64.3±7.9	64.7±7.5	0.542	2.4±8.2	1.5±7.9	0.119
E/Em	12.0±5.3	10.3±4.6	<0.001	12.4±5.9	10.4±4.7	<0.001	0.3±5.0	0.4±3.8	0.907
RVSP, mmHg	27.3±6.9	26.9±7.1	0.507	27.3±8.0	26.0±6.7	0.026	-0.3±8.1	-0.8±7.0	0.362
LVMI, g/m^2	100.9±24.2	93.5±21.7	<0.001	101.1±24.6	92.9±21.7	<0.001	0.5±18.2	-0.3±17.9	0.544
**PVR related parameter**									
Stroke volume	59.0±19.7	58.3±20.3	0.674	64.0±16.5	61.8±18.8	0.109	5.0±21.0	3.4±22.9	0.342
Heart rate, bpm	68.9±14.7	67.0±12.6	0.090	72.6±12.1	71.2±10.8	0.121	4.7±14.5	4.6±13.4	0.921
Cardiac output	4.1±1.3	4.0±1.3	0.191	4.3±1.1	4.3±1.3	0.431	0.2±1.4	0.3±1.5	0.651
PAMP, mmHg	18.6±4.2	18.4±4.3	0.507	18.6±4.9	17.8±4.1	0.026	0.0±4.6	-0.6±4.5	0.085
PCWP, mmHg	16.8±6.5	14.7±5.7	<0.001	17.3±7.3	14.8±5.8	<0.001	0.4±6.4	0.2±4.9	0.744
PVR	0.4±1.7	0.9±1.7	<0.001	0.3±1.6	0.7±1.6	0.003	0.1±1.8	0.3±1.7	0.055

* Data are presented as mean ± SD for continuous variables.

+ LA, left atrium; LVEF, left ventricular ejection fraction; E/Em, the ratio of the early diastolic mitral inflow velocity (E) to the early diastolic mitral annular velocity (Em); RVSP, right ventricular systolic pressure; LVMI, left ventricular mass index; PVR, pulmonary vascular resistance; PAMP, pulmonary artery mean pressure; PCWP, pulmonary capillary wedge pressure.

Supplementary Table 4. Hemodynamic characteristics according to the presence of DM after propensity score matching

	Baseline			1-year follow up			Delta value		
	DM (N=211)	Non-DM (N=633)	p-value	DM (N=211)	Non-DM (N=633)	p-value	DM (N=211)	Non-DM (N=633)	p-value
**Echocardiographic findings**									
LA diameter, mm	43.0±6.0	42.1±6.0	0.063	40.6±5.5	38.8±5.7	<0.001	-2.4±4.4	-3.3±4.8	0.016
LVEF, %	62.0±8.9	63.0±8.5	0.151	64.3±7.9	65.0±7.5	0.247	2.3±7.6	2.0±7.8	0.650
E/Em	12.0±5.3	10.6±4.0	<0.001	12.4±5.9	11.1±4.8	0.005	0.3±5.1	0.5±3.8	0.529
RVSP, mmHg	27.3±6.9	27.2±6.9	0.923	27.3±8.0	26.5±6.7	0.184	0.0±7.5	-0.8±7.1	0.181
LVMI, g/m^2	100.9±24.2	95.2±22.6	0.003	101.1±24.6	95.1±20.6	0.002	0.2±18.3	-0.1±17.8	0.860
**PVR related parameter**									
Stroke volume	50.9±19.7	57.2±19.6	0.256	64.0±16.5	61.5±18.2	0.083	5.0±21.0	4.3±21.3	0.685
Heart rate, bpm	67.9±14.0	66.3±12.7	0.206	72.3±11.1	71.0±10.8	0.184	5.4±13.9	5.2±13.1	0.871
Cardiac output	4.0±1.6	3.9±1.4	0.537	4.6±1.1	4.3±1.3	0.009	0.7±1.7	0.5±1.5	0.202
PAMP, mmHg	18.6±4.2	18.6±4.2	0.923	18.6±4.9	18.1±4.1	0.184	0.0±4.6	-0.5±4.3	0.181
PCWP, mmHg	16.8±6.5	15.0±4.9	<0.001	17.3±7.3	15.7±6.0	0.005	0.4±6.4	0.6±4.7	0.589
PVR	0.5±1.9	1.0±1.6	0.003	0.3±1.5	0.5±1.6	0.174	-0.1±1.8	-0.6±1.6	0.012

* Data are presented as mean ± SD for continuous variables.

+ LA, left atrium; LVEF, left ventricular ejection fraction; E/Em, the ratio of the early diastolic mitral inflow velocity (E) to the early diastolic mitral annular velocity (Em); RVSP, right ventricular systolic pressure; LVMI, left ventricular mass index; PVR, pulmonary vascular resistance; PAMP, pulmonary artery mean pressure; PCWP, pulmonary capillary wedge pressure.

Supplementary Table 5. Logistic regression analysis of the stiff LA physiology in the patients except extra PV LA ablation

	Univariate analysis		Multivariate analysis		Multivariate analysis	
	**OR (95% CI)**	**P value**	**OR (95% CI)**	**P value**	**OR (95% CI)**	**P value**
Age	1.017 (0.953 – 1.084)	0.614				
Male	0.997 (0.296 – 3.358)	0.997				
Paroxysmal AF	0.845 (0.225 – 3.175)	0.803				
Body mass index	1.106 (0.933 – 1.311)	0.245				
Diabetes mellitus	0.621 (0.134 – 2.873)	0.542				
Hypertension	0.830 (0.264 – 2.607)	0.749				
Congestive heart failure	2.426 (0.639 – 9.208)	0.193				
Stroke	0.529 (0.067 – 4.162)	0.546				
Vascular disease	1.032 (0.222 – 4.796)	0.968				
LA diameter	0.994 (0.900 – 1.097)	0.898				
LVEF	1.020 (0.945 – 1.100)	0.615				
E/Em	0.990 (0.862 – 1.137)	0.887				
LA volume/BSA	1.015 (0.989 – 1.042)	0.259				
Pericardial fat volume	1.007 (0.998 – 1.017)	0.121				
LA pressure, peak	1.067 (1.014 – 1.123)	0.012	1.072 (1.013 – 1.134)	0.016	1.062 (1.006 – 1.121)	0.030
LA voltage	0.896 (0.366 – 2.191)	0.809				
LA wall stress	1.004 (0.997 – 1.010)	0.257				
Baseline PVR	1.165 (0.885 – 1.533)	0.277				
1-year follow up PVR	1.722 (1.235 – 2.401)	0.001	1.636 (1.176 – 2.275)	0.003		
Delta PVR	1.542 (1.152 – 2.063)	0.004			1.424 (1.065 – 1.903)	0.017

† Two multivariate models were separately presented because 1-year follow up PVR and delta PVR had a multicollinearity to each other.

‡ LA, left atrium; AF, atrial fibrillation; LVEF, left ventricular ejection fraction; E/Em, the ratio of the early diastolic mitral inflow velocity (E) to the early diastolic mitral annular velocity (Em); BSA, body surface area; PV, pulmonary vein; PVR, pulmonary vascular resistance.

Supplementary Table 6. Logistic regression analysis of the presence of DM in the patients after propensity score matching

	**Univariate analysis**		**Multivariate analysis**		**Multivariate analysis**	
	**OR (95% CI)**	**P value**	**OR (95% CI)**	**P value**	**OR (95% CI)**	**P value**
Age	1.001 (0.983 – 1.019)	0.911				
Male	1.045 (0.745 – 1.466)	0.797				
Paroxysmal AF	0.931 (0.679 – 1.276)	0.657				
Body mass index, BMI	1.074 (1.017 – 1.133)	0.010	1.051 (0.976 – 1.131)	0.186	1.055 (0.980 – 1.137)	0.154
Hypertension	3.150 (2.212 – 4.485)	<0.001	2.178 (1.042 – 2.737)	<0.001	2.247 (1.472 – 3.432)	<0.001
Congestive heart failure	0.856 (0.544 – 1.347)	0.501				
Stroke	1.368 (0.905 – 2.068)	0.137				
Vascular disease	2.208 (1.512 – 3.226)	<0.001	1.689 (1.042 – 2.737)	0.033	1.781 (1.095 – 2.896)	0.020
LA diameter	1.025 (0.999 – 1.052)	0.063				
LVEF	0.987 (0.970 – 1.005)	0.151				
E/Em	1.072 (1.036 – 1.110)	<0.001	0.992 (0.931 – 1.058)	0.813	1.047 (0.995 – 1.101)	0.075
RVSP	1.001 (0.979 – 1.024)	0.923				
LVMI	1.010 (1.003 – 1.017)	0.004	1.003 (0.994 – 1.013)	0.464	1.002 (0.993 – 1.011)	0.641
LA volume/BSA	0.995 (0.988 – 1.001)	0.125				
Pericardial fat volume	1.003 (1.000 – 1.006)	0.036	1.000 (0.996 – 1.004)	0.878	1.000 (0.996 – 1.004)	0.892
LA pressure, peak	1.031 (1.014 – 1.048)	<0.001	1.037 (1.016 – 1.057)	<0.001	1.153 (1.024 – 1.298)	0.019
LA voltage	1.031 (0.817 – 1.301)	0.797				
Extra PV LA ablation	1.125 (0.818 – 1.547)	0.468				
Extra PV trigger	1.210 (0.709 – 2.064)	0.485				
Baseline PVR	0.787 (0.708 – 0.872)	<0.001	0.783 (0.659 – 0.929)	0.005		
1-year follow up PVR	0.910 (0.825 – 1.003)	0.058				
Delta PVR	1.141 (1.033 – 1.261)	0.009			1.033 (1.013 – 1.054)	0.001

* Two multivariate models were separately presented because 1-year follow up PVR and delta PVR had a multicollinearity to each other.

+ AF, atrial fibrillation; LA, left atrium; LVEF, left ventricular ejection fraction; E/Em, the ratio of the early diastolic mitral inflow velocity (E) to the early diastolic mitral annular velocity (Em); LVMI, left ventricular mass index; BSA, body surface area; PV, pulmonary vein; PVR, pulmonary vascular resistance

Supplementary Table 7. Cox regression analysis for clinical recurrence of AF after catheter ablation

	Univariate analysis		Multivariate analysis	
	**HR (95% CI)**	**P value**	**HR (95% CI)**	**P value**
Age	1.009 (0.997 – 1.022)	0.144		
Male	0.996 (0.789 – 1.258)	0.976		
Paroxysmal AF	0.500 (0.402 – 0.623)	<0.001	0.781 (0.558 – 1.092)	0.149
Body mass index	1.024 (0.987 – 1.063)	0.208		
Diabetes mellitus	0.888 (0.687 – 1.149)	0.367		
Hypertension	1.016 (0.813 – 1.268)	0.891		
Congestive heart failure	1.332 (0.996 – 1.782)	0.053		
Stroke	0.944 (0.696 – 1.279)	0.708		
Vascular disease	1.075 (0.815 – 1.416)	0.610		
LA diameter	1.044 (1.025 – 1.063)	<0.001	1.006 (0.980 – 1.033)	0.641
LVEF	0.991 (0.980 – 1.003)	0.155		
E/Em	1.012 (0.988 – 1.037)	0.330		
LA volume/BSA	1.013 (1.009 – 1.017)	<0.001	1.011 (1.006 – 1.017)	<0.001
Pericardial fat volume	1.001 (0.999 – 1.003)	0.161		
LA pressure, peak	1.008 (0.997 – 1.019)	0.173		
LA voltage*	0.577 (0.481 – 0.694)	<0.001		
LA wall stress*	1.001 (1.000 – 1.003)	0.006	1.000 (0.999 – 1.002)	0.885
Extra PV LA ablation	1.791 (1.439 – 2.230)	<0.001	1.213 (0.871 – 1.689)	0.253
Extra PV trigger	1.600 (1.125 -2.275)	0.009	1.578 (1.084 – 2.297)	0.017
Stiff LA physiology	1.691 (1.038 – 2.757)	0.035	1.358 (0.727 – 2.539)	0.337

* LA wall stress was included in the multivariate analysis due to multicollinearity among three variables.

+ LA, left atrium; AF, atrial fibrillation; LVEF, left ventricular ejection fraction; E/Em, the ratio of the early diastolic mitral inflow velocity (E) to the early diastolic mitral annular velocity (Em); BSA, body surface area; PV, pulmonary vein; PVR, pulmonary vascular resistance.
